# Mitogenome selection in the evolution of key ecological strategies in the ancient hexapod class Collembola

**DOI:** 10.1038/s41598-022-18407-1

**Published:** 2022-08-31

**Authors:** Daniela M. Monsanto, Devon C. Main, Charlene Janion-Scheepers, Arsalan Emami-Khoyi, Louis Deharveng, Anne Bedos, Mikhail Potapov, Shilpa P. Parbhu, Johannes J. Le Roux, Peter R. Teske, Bettine Jansen van Vuuren

**Affiliations:** 1 Centre for Ecological Genomics and Wildlife Conservation, Department of Zoology, University of Johannesburg, Auckland Park, 2006 South Africa; 2 Department of Biological Sciences, University of Cape Town, Rondebosch, 7701 South Africa; 3 Iziko South African Museum, Gardens, Cape Town, 8001 South Africa; 4 Institut de Systématique, Evolution, Biodiversité (ISYEB)-UMR 7205-CNRS, UPMC, EPHE, Muséum National d’Histoire Naturelle (MNHN), Sorbonne Université, 75231 Paris, France; 5 Department of Zoology and Ecology, Institute of Biology and Chemistry, Moscow State Pedagogical University, Moscow, 119991 Russia; 6 Senckenberg Museum of Natural History, 60325 Görlitz, Germany; 7 School of Natural Sciences, Macquarie University, Sydney, NSW 2109 Australia

**Keywords:** Phylogenetics, Molecular evolution

## Abstract

A longstanding question in evolutionary biology is how natural selection and environmental pressures shape the mitochondrial genomic architectures of organisms. Mitochondria play a pivotal role in cellular respiration and aerobic metabolism, making their genomes functionally highly constrained. Evaluating selective pressures on mitochondrial genes can provide functional and ecological insights into the evolution of organisms. Collembola (springtails) are an ancient hexapod group that includes the oldest terrestrial arthropods in the fossil record, and that are closely associated with soil environments. Of interest is the diversity of habitat stratification preferences (life forms) exhibited by different species within the group. To understand whether signals of positive selection are linked to the evolution of life forms, we analysed 32 published Collembola mitogenomes in a phylomitogenomic framework. We found no evidence that signatures of selection are correlated with the evolution of novel life forms, but rather that mutations have accumulated as a function of time. Our results highlight the importance of nuclear-mitochondrial interactions in the evolution of collembolan life forms and that mitochondrial genomic data should be interpreted with caution, as complex selection signals may complicate evolutionary inferences.

## Introduction

Understanding the interplay between genomes, morphology, functional ecology, and biogeography in driving organismal evolution has become central to answering fundamental questions in biology, both from historical (e.g. past speciation) and contemporary (e.g. evolutionary responses to climate change) viewpoints. Making sense of the immense complexity of life has proven a recalcitrant challenge to biologists. Additional lines of evidence pertaining to taxonomy (the fossil record in conjunction with morphological, behavioural, physiological, and molecular data) enhance our ability to piece together the diversification of life on Earth^1–4^. A central question in molecular evolution revolves around how natural selection influences the genomic architectures of organisms^5–10^. Environmental pressures drive functional and morphological changes, which are underpinned by genetic variation and, therefore, will manifest as signatures of selection on the genome that can be used to retrace the evolutionary history of organisms and better predict future adaptive evolutionary trajectories.

The mitochondrial genome comprises 13 protein-coding genes (PCGs), which, together with many nuclear genes, encode the proteins that make up the subunits of the four complexes involved in the oxidative phosphorylation chain^11,12^. Due to the crucial role of PCGs in the respiratory chain, these genes are functionally highly constrained^5^. An evaluation of signals of past selection on these genes, therefore, provides insights into the adaptive evolution and metabolic requirements of organisms, given that they are responsible for 95% of the energy metabolism and heat production in cells^11,13,14^. Mitochondrial genes are some of the most commonly used genetic markers, partly because they provide unique perspectives on population genetic variation and structure due to maternal inheritance and a general lack of recombination^15–17^, and are frequently used to answer questions related to historical processes and geographical structure^13,14,18,19^. However, these inferences often assume that the evolution of this genome is neutral^20,21^, an idea that has been challenged^22–26^. It is now widely accepted that the mitogenome is under selection due to its pivotal role in respiration and energy production, and the evolutionary constraints resulting from the co-evolution of nuclear and mitochondrial genes that are involved in these processes^11,12,15–17,25^. Additionally, positive selective sweeps, linked to thermal and aerobic respiratory adaptations, are well documented for mitochondrial genes across various taxa^11,13,14,27–32^. However, the investigation of positive selection on moderately conservative genomic regions, without an assessment of the functional consequence of amino acid mutations, is not adequate to consider the mitochondrial genome in its entirety to be under positive selection linked to adaptive processes^13^. Therefore, we identify both signals of positive selection and the functional and adaptive significance of amino acid mutations on the mitochondrial genomes of ancient soil-dwelling invertebrate taxa.

Given the influence of heterogeneous soil environments on soil biota community assemblages^33–35^, and that positive selection on the mitogenome is linked to thermal and aerobic respiratory adaptations, we sought to explore the effects of signatures of past selection on the mitogenome of one of the most ancient soil-dwelling invertebrate taxonomic groups, Collembola. Commonly referred to as springtails, this group of primitive, wingless, and largely terrestrial hexapods is regarded as among the first aquatic arthropods to have colonised land^36^, with evolutionary roots that predate the diversification of insects^37–40^. Indeed, the earliest known hexapod fossil (from the early Devonian period, ca. 400 Ma) is a collembolan^41,42^. This group's biology and ancient evolutionary history provide unique opportunities to understand the environmental and physiological mechanisms governing the evolution of early terrestrial life. Collembolans currently exploit a myriad of niches across all continents, including Antarctica^40,43,44^. They typically reside within, or are closely associated with soils, and are considered essential components of soil ecosystems due to their influence on soil microbial communities, their role in nutrient recycling, and their importance as prey to a wide range of predatory arthropods^45–48^. Of particular interest is the preference of various springtails for different habitats. Springtail species broadly fall into six different ecological life form categories, each displaying a unique stratification preference. These are atmobiotic (large species that inhabit macrophytes (grasses, bushes, tree trunks and branches)); epiedaphic (species found in vegetated habitats or the upper litter layer above the soil); hemiedaphic (which includes species that occupy decomposed litter or rotten wood on the soil surface); euedaphic (‘true’ soil living species that occupy the upper mineral-rich layers of the soil); myrmecophilous (describes species found in ant nests), and hydrophilous (hereafter referred to as aquatic for simplicity; species that live on the surfaces of, or are closely associated with, freshwater bodies)^34,43,47,49,50^.

Strong correlations exist between the life forms of springtails and their morphologies, suggesting that life forms are subject to selective forces exerted by their environment^43,45–47,51–53^. Additionally, springtails also exhibit highly specific habitat preferences for different soil types, soil chemistries, habitat types, and stratification within a given habitat^47,49,52^. It is remarkable that these morphological designs have probably persisted unchanged for millions of years in many instances. Indeed, springtail fossils dating back as far as the Eocene (ca 40–50 Ma) can be assigned to extant genera and, in some cases, even extant species^54^. This extreme morphological conservatism suggests that current morphospecies inventories underestimate true collembolan diversity. Not surprisingly, genetic evidence has shown that cryptic diversity is pervasive in this group, with current collembolan species richness thought to underestimate the actual number of species by at least an order of magnitude^40,55,56^. Therefore, the low number of known springtail species is not an inherent characteristic of the group, but rather the result of under sampling, outdated taxonomy, and a lack of taxonomic expertise^56^. While Collembola may be dwarfed by other hexapod groups, particularly insects, it still harbours immense diversity^40^.

Given that Collembola constitutes ecologically important taxa within soil environments^48^, which exhibit distinct microhabitat stratification that is driven by feeding strategies, food availability, and soil type and chemistry^47,49,50,52,57^, we sought to identify mitochondrial genes that are under positive selection for adaptations to these microhabitats. Additionally, given that metabolic activity and oxygen consumption differ depending on ecological life form^58,59^, we investigated whether signals of selection on the mitochondrial genome are associated with the evolution of life form traits (habitat preferences), in the context of environmental change (oxygen levels/metabolic requirements and vegetation alterations) that emerged during the group's evolutionary history. If this is the case, we expect substantial signals of selection to have accumulated along phylogenetic branches representing shifts in life forms compared to sister branches where the original life form was maintained.

## Results

### Dated phylogeny and ancestral state reconstruction

Our dated mitogenomic phylogeny demonstrates that Collembola first diverged sometime in the early Devonian (ca. 410 Ma), with most diversification taking place in the Triassic to Cretaceous periods (ca. 250–60 Ma) (Fig. S1).

**Figure 1:**
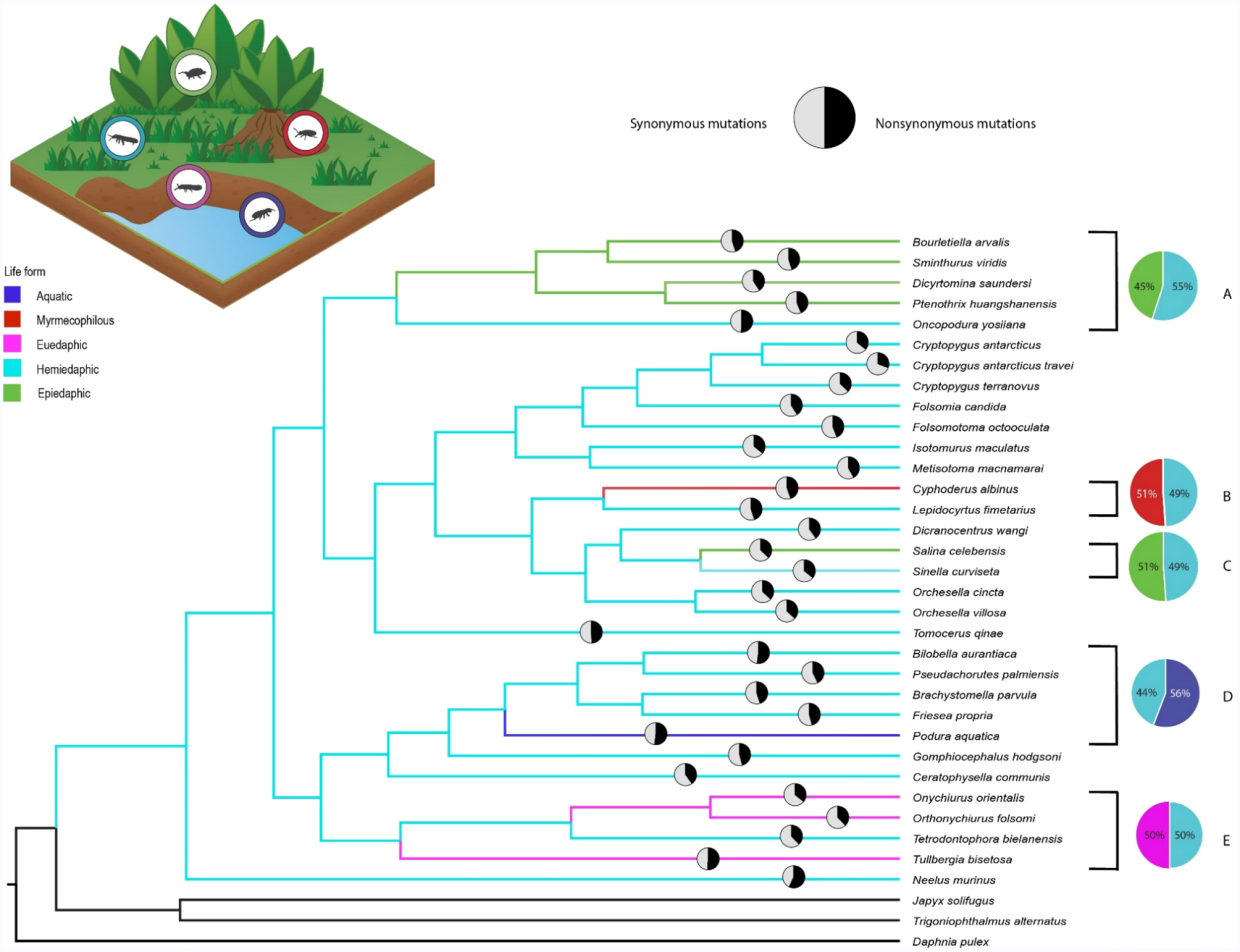
Bayesian phylogenetic tree based on the mitogenomes of 32 collembolan species from four orders and three outgroup taxa indicating the evolution of life form traits for each species (blue: aquatic; red: myrmecophilous; pink: euedaphic; cyan: hemiedaphic, and green: epiedaphic). The pie charts along the terminal branches represent the number of nonsynonymous (black segments) and synonymous (grey segments) mutations. The coloured pie charts represent the proportion of nonsynonymous mutations (i.e. dN/(dN + dS)) for the branches/clades that represent the evolution of an alternative life form (blue: aquatic; red: myrmecophilous; pink: euedaphic, and green: epiedaphic) vs. the sister branches/taxa that represents the ancestral state (cyan: hemiedaphic). Where multiple branches are present (i.e. clades), we accounted for the number of taxa by averaging the proportion of mutations for each terminal and internal branch.

To identify whether a link exists between signals of mitochondrial DNA selection and collembolan life form (see Dataset S1), we assessed phylogenetic relationships between 32 collembolan and three outgroup taxa (see Table S1). We assigned a life form trait (i.e. aquatic, epiedaphic, euedaphic, hemiedaphic, and myrmecophilous) to the ecology (i.e. habitat preference) of each springtail species included in this study (see Dataset S1 for more detail). We reconstructed the ancestral ecological life form state for the collembolan clade using three different methods (parsimony, maximum likelihood, and Bayesian inference), all of which identified hemiedaphic as the ancestral character (see Fig. 1). Across the collembolan phylogeny, five independent life form shifts occurred from the hemiedaphic to alternative life forms (see Fig. 1). For the terminal nodes, the most common ecological life form was hemiedaphic (22 taxa), followed by epiedaphic (five taxa), euedaphic (three taxa), myrmecophilous (one taxon), and aquatic (one taxon). To assess phylogenetic conservatism and homoplasy across the phylogeny, we calculated the retention index (RI)^60^ for each life form trait, which was 0.75 for epiedaphic, 0.36 for hemiedaphic, 0.33 for euedaphic, and 0 for both myrmecophilous and aquatic.

### Selection analysis

**Figure 2:**
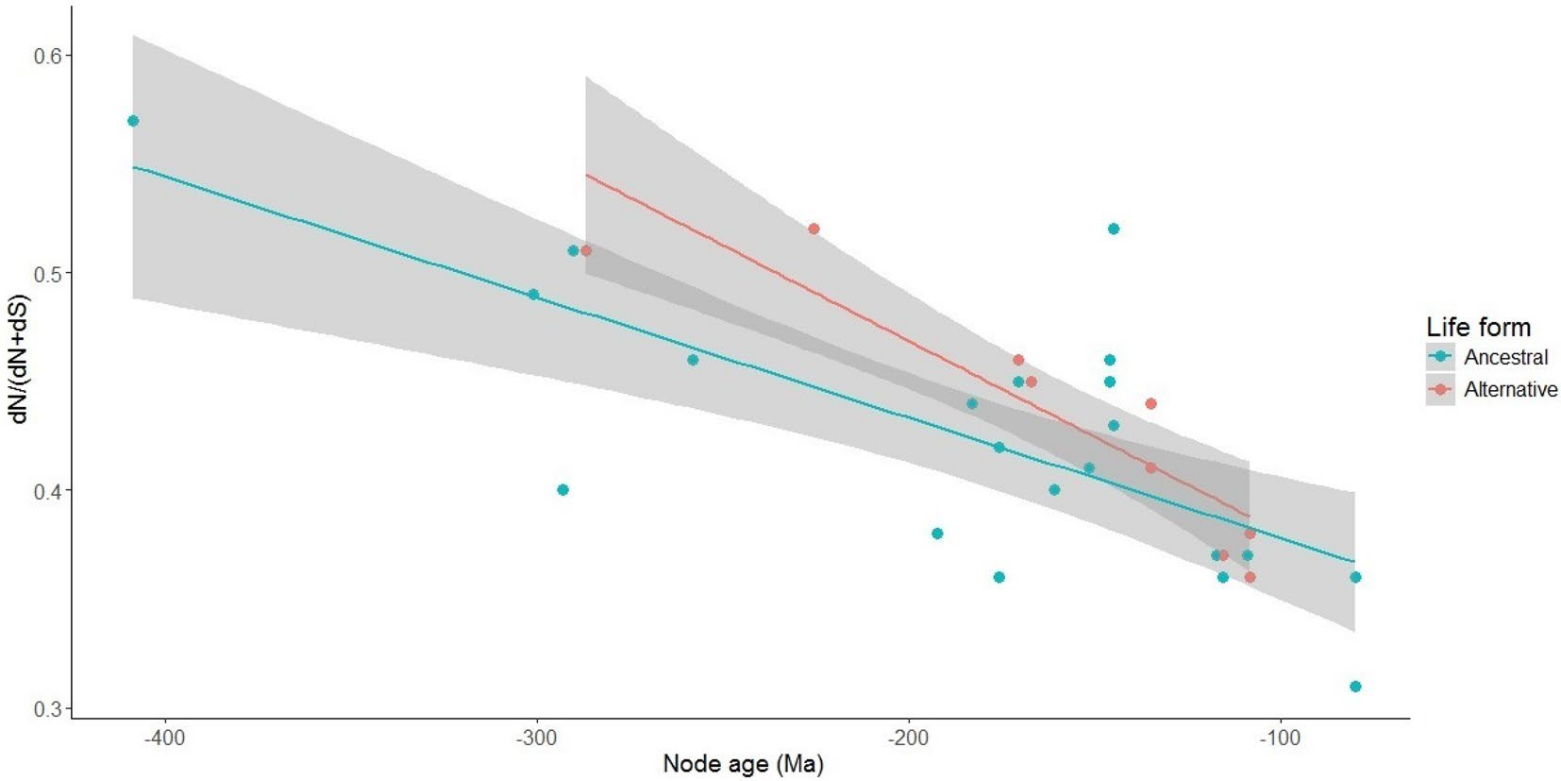
Scatter plots and regression lines displaying the number of nonsynonymous mutations as a proportion of the total number of mutations (i.e. dN/(dN + dS)) as a function of time (i.e. branch length/node age (Ma)). The blue dots represent the proportion of nonsynonymous mutations for taxa with the ancestral state (R^2^ = 0.52), while the red dots represent the taxa that illustrate life form shifts (i.e. the alternative state) (R^2^ = 0.82). The shaded regions represent the 95% confidence intervals of the regression lines.

To assess signals and direction of selection at different loci (i.e. positive vs purifying selection), we used the codon-based maximum likelihood (CodeML) algorithm to determine the ratio of nonsynonymous to synonymous mutations (ω = dN/dS), and the number of nonsynonymous mutations as a proportion of the total number of mutations (dN/(dN + dS))^61–64^. Site model selection analyses indicated overall positive selection across the phylogeny, shown by significant likelihood ratio tests (LRT) for the comparative site models, however, with no nucleotide positions under positive selection (Bayes Empirical Bayes score (BEB) < 0.95^62^) (Table S2). The comparison between the numbers of synonymous to nonsynonymous mutations across the terminal nodes of the phylogeny was significantly different (paired *t* test: *t*_415_ = − 9.48, *p* < 0.05). The number of nonsynonymous mutations as a proportion of the total number of mutations revealed no strong evidence for a link between life form transitions and the number of nonsynonymous mutations. Departures from the expectation that synonymous mutations are more common than nonsynonymous mutations^65^ were found in several taxa (e.g. *Bilobella aurantiaca*, *Neelus murinus*, *Oncopodura yosiiana*, *Podura aquatica*, and *Tullbergia bisetosa*) (see black and white pie charts in Fig. 1 and Table S3 for proportion values > 0.50). However, only the life histories of *Podura aquatica* and *Tullbergia bisetosa* deviated from the hemiedaphic ancestral state. The large proportions of nonsynonymous mutations are likely due to the older ages of these branches and illustrate that the proportion of nonsynonymous mutations increases as a function of time for the taxa with the ancestral state compared to those that represent life form shifts (i.e. the alternative state) (ancestral state: R^2^ = 0.52; alternative state: R^2^ = 0.82; also see Fig. 2). The 95% confidence intervals of these regressions broadly overlap, and the two models were not significantly different (*p* > 0.05). This indicates that no significant difference was found in the proportion of nonsynonymous mutations in relation to branch length for taxa that retained the ancestral state and for those whose ecological life form shifted.

The relationships (represented as coloured pie charts in Fig. 1) between the taxa that represent the life form transitions to alternative life forms and the respective sister taxon/taxa (ancestral state, i.e. hemiedaphic), again revealed that the longer branches have the greatest number of nonsynonymous mutations in comparison to the sister clades (see coloured pie charts A and D). No significant difference was found within pie charts B, C, and E, which show that the proportion of mutations for the branches that represent the alternative life form vs. ancestral state (51% for pie charts B and C, and 50% for pie chart E; paired *t* test: *t*_4_ = 0.23, *p* > 0.05) (Fig. 1).

Evidence for purifying (ω < 1) or diversifying selection (ω > 1) was determined for each of the terminal branches, and a total of 19 taxa had mitogenomes with signatures of purifying selection, while the mitogenomes of 11 taxa showed signs of diversifying selection, and two taxa had mitogenomes that satisfied the assumptions of neutrality (see Table S3). The proportion of changes per gene and per taxon reveal that *atp8* exhibits the highest number of changes for the majority of the taxa (Figs. S2, S3; Table S4). Following *atp8*, all genes from complex I (i.e. *nad1*–*nad6*) had a large proportion of nonsynonymous mutations. Of the 13 PCGs, eight genes were identified as experiencing purifying selection, while five genes were identified as experiencing diversifying selection, with *atp8* being under particularly strong diversifying selection (ω > 3.0) (Table S4). At a complex level, complex V (ATPase) revealed the highest percentage of nonsynonymous mutations, followed by complex I (NDs), complex IV (COXs), and complex III (*cytb*) (Fig. S3), which follows the estimated mutation rate of these complexes (i.e. ATPase > ND > COX > CYTB)^66^. The average value of ω for all genes across all terminal nodes was 0.96, which indicates that, overall, Collembola mitogenomes are experiencing slight purifying selection or no selection (i.e. selective neutrality).

### Radical changes in physicochemical properties of amino acids

The impact of positive selection on the physical and chemical properties of 31 amino acids was evaluated for each of the 13 PCGs by detecting observed changes inferred by the phylogenetic tree under neutral conditions. Both positive and negative significant z-scores on the physicochemical properties of amino acids were found across the tree. Most physicochemical properties indicated that the radical changes were under purifying selection, with occasional radical changes under positive selection (Fig. S4). We detected two physicochemical properties—equilibrium constant (ionisation of COOH) and solvent accessible reduction ratio—with significant radical changes (z > 3.09; *p* < 0.001), indicating positive/diversifying selection for all 13 PCGs, while a third physicochemical property, buriedness, is under positive/diversifying selection for 10 PCGs (except *cytb*, *nad3*, and *nad4l*), with *atp8* having the highest z-score.

## Discussion

Using a comprehensive mitogenome dataset for springtails (32 species from all four orders), we determined the ancestral ecological life form state for Collembola. We then used this dataset to assess whether a correlation exists between signatures of mitogenome selection and the evolution of novel life forms. We show that the ancestral ecological life form state of Collembola is hemiedaphic, with epiedaphic life forms first emerging in the early Permian, roughly 290 Ma. Even though several previous studies have identified links between Collembolan mitogenomes and ecology, morphology, and physiology^50,53,67–71^, we found no strong evidence for a link between signatures of mitochondrial genome selection and life form evolution in Collembola.

Our mitogenomic phylogeny is broadly consistent with those presented in earlier studies^37–39,72–75^. The first phylogenetic split within Collembola occurred during the early Devonian (ca. 410 Ma), with most diversification taking place in the Triassic to Cretaceous periods (ca. 250–60 Ma). It is not surprising, therefore, that Collembola fossils preserved in amber and dating back to the Eocene (50-40 Ma) can be assigned to extant taxa with high certainty^54^, demonstrating the ultraconservative nature of life forms and basic body plans of the group.

Ancestral state reconstructions showed the ancestral ecological life form of collembolans to be hemiedaphic. Our finding contrasts with the assumption of a euedaphic origin of springtails proposed by D’Haese^36,76^. Additionally, we found that the epiedaphic life form evolved at least twice, first during the origin of Symphypleona (Fig. S1), approximately 290 Ma, and then again in the mid-Cretaceous with the emergence of the evolutionary lineage represented by *Salina celebensis*, roughly 115 Ma. During the Permian period, roughly 300 Ma, seed-producing gymnosperms such as gnetophytes, Pinaceae, and cupressophytes diverged from cycads and conifers^77^. Symphypleona show a habitat preference for Mediterranean-type shrublands, pine plantations, mixed forests, bushes, grasslands, and leaf litter (see Supplementary Information Dataset S1). This suggests that the emergence of major plant lineages, such as gymnosperms, created new ecological niches for the epiedaphic Symphypleona to exploit and diversify. The extant *Bourletiella arvalis* and *Sminthurus viridis*, however, show a habitat preference for grasslands, although these habitats would have only become available much later in the Cenozoic^78–80^. It is, therefore, reasonable to assume that the evolutionary lineage represented by these species would have occupied gymnosperm ecological analogs until their current niches became available. Unsurprisingly, the morphological features of these taxa are characteristic of the epiedaphic life form^47,49,81^. Additionally, our analyses illustrate that the euedaphic life form evolved at least twice, first when *Tullbergia bisetosa* evolved, roughly 287 Ma, and secondly during the origin of *Onychiurus orientalis* and *Orthonychiurus folsomi* (approximately 192 Ma). A shift from hemiedaphic to the myrmecophilous and aquatic life forms was also identified in the phylogeny. The origin of *Cyphoderus albinus* and the myrmecophilous life form occurred approximately 170 Ma, coinciding with the evolution of ants (Formicidae) around 168 Ma^82,83^. The aquatic life form first emerged about 225 Ma with the divergence of the lineage represented by *Podura aquatica*. The hypothesis of a semi-aquatic or aquatic life form as the ancestral state of Collembola was formulated and tested by D’Haese^36,76^, who found that the evolution of terrestrial to aquatic habitats, in a physiological context, is indeed more plausible and that an aquatic life form can be viewed as a “rare secondary acquisition”^36,43,76^.

We deem the five life forms we have identified to be somewhat homoplastic (see Yu et al.^53^) best illustrated by the epiedaphic and euedaphic forms that independently originated twice within the group. Additionally, the myrmecophilous and aquatic forms are homoplastic or autapomorphic, as these traits are unique to single taxa. There is also a great deal of ecological conservatism for the hemiedaphic trait where most extant taxa have retained the ancestral state.

While there appeared to be a general trend for taxa on longer phylogenetic branches to have a greater overall proportion of nonsynonymous mutations, we found no consistent relationship between life form shifts and the proportion of nonsynonymous mutations on these branches. Furthermore, even when comparing individual genes, no correlations were found between the proportion of nonsynonymous changes and the evolution of new life forms (Fig. S2). This may seem surprising given the pivotal role that mitochondria play in aerobic metabolism, and given that different life forms are constrained in different ways metabolically. It should be noted, however, that for a group as ancient as Collembola, the many adaptive nuclear changes that produced the conspicuous ecological changes upon which life forms are classified may have obscured mitochondrial signatures of selection related to the evolution of life forms. While our expectations pertaining to selection were premised on non-overlapping habitat stratification preferences, it should also be noted that interactions between collembolans and their environments are complex, and apparently divergent life forms may impose similar functional constraints on the organism, thereby confounding signatures of selection^84^.

Even though mitochondrial protein coding genes are typically believed to have faster mutation rates than nuclear genes^16^, most of these mutations are probably synonymous^85,86^, and thus functionally inconsequential (but see Lawrie et al.^87^). Our results support this idea, with more taxa and more genes under purifying selection than under diversifying selection (Tables S3 and S4). However, selection on the mitochondrial genome appears to be largely neutral due to the random fixation of selectively neutral mutations^21^. Given the crucial role of mitochondria in aerobic metabolism, functional requirements likely constrain the molecular evolution of mitogenomes, and this is perhaps why many studies have identified purifying selection^88–90^. It makes sense that strong functional constraints would mean that any deleterious mutation that arises would be rapidly removed from the population via purifying selection. Additionally, genetic drift plays a major role in structuring genetic diversity, and its effects on mitochondrial genomes are stronger than on nuclear genomes due to differences in effective population sizes between them and the uniparental inheritance and lack of recombination within the mitochondrial genome^16,17,21^.

When radical amino acid changes are favoured by selection, local directional shifts in the structure and function of proteins evolve. The physicochemical properties under positive selection for all PCGs were equilibrium constant (ionisation of COOH) and solvent accessible reduction ratio, while buriedness was under positive selection for 10 of the 13 PCGs. Positive selection for equilibrium constant is suggestive of adaptations to increased oxygen levels and metabolic requirements^66^, while positive selection for solvent accessible reduction ratio may lead to bulkier proteins, which increases the space for active site formation^66^. Buriedness increases protein stability, and thus optimal protein function, in the context of hydrophobicity, whereby some residues are buried on the interior of the globular protein^91,92^. These results illustrate the importance of these properties in the context of environmental conditions (oxygen levels and metabolic requirements).

The mitochondrial genome has long been considered to be selectively neutral^22–25^, but a plethora of studies have illustrated that it may be under strong positive selection as a result of thermal and aerobic respiratory adaptations^11,13,14,27–32^. Here, we investigated whether a link exists between mitochondrial genome selection and the evolution of ecological life forms in Collembola. The expected outcome of such a link would be evidence for strong signals of selection at branches representing the evolution of new life forms, even if mitochondrial genes themselves are not the primary drivers of ecophysiological adaptation. Our selection results reveal that, overall, positive selection is present across the collembolan phylogeny, best illustrated by the presence of positive selection on three amino acid physicochemical properties which likely play instrumental roles in adaptive responses to environmental conditions (oxygen levels and metabolic requirements). However, the only major trend identified here was that the proportion of nonsynonymous mutations increased with time, with longer branches (i.e. older taxa) having a greater proportion of nonsynonymous mutations than shorter branches. In addition to investigating selection associated with life form evolution, we assessed whether there was a link between signatures of selection across a latitudinal gradient from where each species can be found. This was done in two ways; first by selecting species across all latitudes but where their ranges overlapped, and secondly by selecting species that had no overlapping distributions. As was the case for life form shifts, we found no clear correlation between the number of nonsynonymous mutations and latitudinal gradients for either analysis.

The lack of a correlation between life form shifts and mitogenome selection may hint at nuclear selection, and suggests that nuclear-mitochondrial interactions are of greater importance in the evolution of life form shifts than selection pressures on the mitochondrial genome alone. Our findings raise some precautionary flags in that selection, where present, is more complex than generally assumed, given the complex interactions of the mitochondrial and nuclear genomes. Importantly, evidence for strong selection on the mitochondrial genome cannot simply be extrapolated to reflect adaptation to changing environmental conditions.

## Methods

### Phylomitogenomic analysis

Phylomitogenomic relationships between Collembola taxa based on available data on GenBank (May 2020) were reconstructed using the protein-coding genes (PCGs) of 32 partial or complete Collembola mitogenomes, as well as three outgroup taxa (see Supplementary Information Table S1). Each PCG was aligned using the MUSCLE algorithm in MEGA X^93^, followed by concatenating the alignments into a single dataset. Substitution saturation was assessed on each codon position using DAMBE v7.3.5^94^, which revealed high substitution saturation, likely due to highly divergent taxa^95–97^. As a result, we took a conservative approach and used the amino acid sequences of each PCG for our phylogenetic reconstructions. Each PCG was independently translated into amino acid sequences using the invertebrate mitochondrial genetic code, and were then aligned in MEGA X using the MUSCLE algorithm, followed by manual inspection. Bayesian phylogenetic inferences were executed in BEAST v2.6.3^98^ by linking site and clock models as well as trees. This was done because of the largely uniparental inheritance of mitochondrial genomes and a lack of recombination^15–17,27^. A discrete trait was added to define the ecological life form of each species (see Supplementary Information Dataset S1 for more detail). Given the role of mitochondria in different environments, and rather than linking life forms to morphological characteristics, we linked the life forms (i.e. aquatic, myrmecophilous, euedaphic, hemiedaphic, and epiedaphic) to the ecology (i.e. habitat preferences) of each springtail species included in this study however, with the exception of three taxa; *Onychiurus orientalis*, *Orthonychiurus folsomi*, and T*ullbergia bisetosa*, which were assigned based on morphology. These species, although ecologically hemiedaphic^47,49,50^ (see Dataset S1 for ecological life forms), differ from other hemiedaphic taxa by having all three major euedaphic morphological characteristics (no eyes, no pigment, and no furca), and are thus categorised as “euedaphomorphic” sensu lato^99^. As such, these taxa were assigned to the euedaphic life form which aligns to a peculiar ecology within litter (i.e. lower dispersal ability and associated traits) that differs from the other hemiedaphic taxa. The mitochondrial REV + Г model was selected, with an uncorrelated relaxed lognormal clock and a birth–death tree prior. Monophyly was constrained for several clades based on a consensus tree constructed using the NCBI taxonomy database in PhyloT v2 (https://phylot.biobyte.de/), and visualised in iTOL^100^. These were (a) all taxa (to calibrate the root of the tree, as described below), (b) the Hexapoda clade (all taxa excluding the distantly related *Daphnia pulex*) to calibrate the split between Collembola and other basal hexapod taxa (*Japyx solifugus* and *Trigoniophthalmus alternatus*) and Collembola as described below, and (c) a clade that excluded all non-Collembola taxa. Monophyly was not constrained for each collembolan order (Poduromorpha, Entomobryomorpha, Neelipleona, and Symphypleona) since monophyly is not always supported for these groups^101–103^.

Considering that some collembolan fossils have been assigned to contemporary species despite being more than 20 Ma old, dating recent nodes using fossil data can be challenging for these taxa. We therefore used secondary calibration points from Leo et al.^104^ and included calibration points that matched the two deepest fossil-calibrated nodes. To ensure that our calibration points matched the 95% highest probability density (HPD) confidence intervals of Leo et al.^104^ for the same nodes, we followed their protocol in setting the priors for these nodes. Accordingly, we set the prior at the root of the tree to have a normal distribution and a mean of 510 Ma with a standard deviation of 7 Ma. The second prior was placed at the split between the higher insects (*Japyx solifugus* and *Trigoniophthalmus alternatus*) and Collembola, with a normal distribution and a mean of 485 Ma with a standard deviation of 6 Ma (for the rationale behind this prior assignment, see Leo et al.^104^).

A Bayesian consensus phylogenetic tree was calculated using BEAST submitted through the CIPRES Science Gateway^105^ from three independent chains at different starting seeds for a chain length of 280 million generations, a thinning interval of 10,000, and 25% burn-in to ensure that stationarity was reached. Convergence was assessed using Tracer v1.7^106^, trees were summarised using TreeAnnotator v2.6.2^98^ (20% burn-in), and the resulting topology was visualised in FigTree v1.4.3 (http://tree.bio.ed.ac.uk/software/figtree/).

### Ancestral state reconstruction

A quantitative phyletic approach was used to assess the evolutionary relationships between taxa based on this biological information. Ancestral state reconstructions were performed using both parsimony and maximum likelihood methods in Mesquite v3.61^107^; the discrete trait of each taxon (from Supplementary Information Dataset S1) was provided as an input for the analyses. The likelihood ratio test of the maximum likelihood method was used for the Markov k-state one-parameter (Mk1) and the asymmetrical Markov k-state 2 parameter (AsymmMk) models^107,108^, while the rate of character evolution was estimated under the Mk1 model. The Bayesian consensus phylogenetic tree obtained from the 28,000 trees (after the burn-in) sampled from BEAST was used for the ancestral state reconstruction. The retention index (RI)^60^ was evaluated for each life form trait to assess phylogenetic conservatism and homoplasy across the phylogeny. High RI values represent little to no homoplasy (typically above 0.85, see Yu et al.^53^), while low RI values indicate homoplasy and low phylogenetic signal in a given dataset.

To account for uncertainties with phylogenetic mapping and the occurrence of topographical incongruence across sampled trees, a Bayesian MCMC method implemented in BayesTraits v3.0.2^109^, was used for ancestral state reconstructions. The *AddTag *and* AddMRCA* options were implemented to find the ancestral state associated with the node of collembolan clade. These options were used to overcome uncertainty when estimating ancestral states^109^. A reversible-jump (RJ) hyper-prior was implemented to reduce uncertainty and arbitrariness when selecting priors^109^. The hyper-prior draws up values from a uniform distribution between 0 and 30 which is used to seed the exponential distribution for the MCMC analyses. Three independent MCMC chains were run for 30 million iterations with a 25% burn-in. These analyses were repeated with a gamma reversible-jump hyper-prior (mean: 0, 30; variance 0, 30), but no appreciable difference was identified between the posterior distributions between these analyses when visualised in Tracer. MCMC convergence was assessed in Tracer by ensuring that the effective sample size (ESS) ≥ 200 for all estimated parameters. The proportion of each state was calculated from the mean values from approximately 20,000 trees randomly chosen by BayesTraits from the 28,000 trees sampled from BEAST. The states that were reconstructed to be equivocal (probability of 0.2 for each state) were discarded and the probability that the collembolan clade was in a given state was determined. The average of the estimated probability for the three independent runs was calculated, and any character state with a proportion of > 0.7 was considered to be supported^110^.

### Selection analysis

The ratio of nonsynonymous, (dN; i.e. amino acid-altering) to synonymous (dS; i.e. silent) substitution rates, ω = dN/dS, is used to understand the evolutionary dynamics of genes^61–64^. We computed ω using the codon-based maximum likelihood (CodeML) algorithm in EasyCodeML v1.21^62,111^. To evaluate the influence of selection on the PCG sequence alignments, we implemented the preset (nested models) running mode with the site model within the framework of the topology of the phylogeny retrieved by the phylogenetic analyses above^112^. The codon substitution models described by Geo et al.^62^, and the significance of their respective dN/dS ratios, were evaluated using the likelihood ratio test (LRT). To identify whether a link between signatures of mitochondrial selection and a shift in ecological life form exists, we determined the number of nonsynonymous mutations per gene as a proportion of the total mutations (i.e. dN/(dN + dS)) for all terminal branches, and then averaged these proportions per branch. Moreover, we compared the proportion of nonsynonymous mutations for each life form shift (aquatic, myrmecophilous, euedaphic, and epiedaphic) vs. the respective sister branches/taxa that represented the ancestral state (hemiedaphic) to ascertain whether a link between the number nonsynonymous mutations to the ecological life form transition exists. Where multiple branches were present (clades), we accounted for the number of taxa by averaging the proportion of mutations for each terminal branch, and then followed the path from the terminal branch back until reaching the node where life form divergence (i.e. nearest common ancestor) took place. Statistical analyses were performed using a paired *t* test to compare the proportion of synonymous to nonsynonymous mutations across the phylogeny as well as where each branch is paired with its sister branch that represents a life form shift. Plots of regression analyses were performed using the R package ggplot2^113^ to assess the correlation between the proportions of nonsynonymous changes vs. branch length for the taxa that retained the ancestral state and for those who represent a life form shift (i.e. the alternative state). Univariate general linear models of the regressions were performed in SPSS 27 (Inc., Chicago, IL, USA). Furthermore, and following the same method as described above, we assessed whether there was a link between selection and species that originated from different latitudinal gradients. This was done twice, first by identifying species across latitudinal gradients where their ranges overlapped, and secondly by selecting species that had no overlapping distributions. A phylogenetic tree was reconstructed for both analyses, and selection analyses were conducted, and the number of nonsynonymous mutations were determined for each terminal branch/species.

Additionally, we sought to determine the evolutionary dynamics in the context of purifying or diversifying selection for each terminal branch based on the count of nonsynonymous and synonymous mutations (i.e. ω) for each gene and complex. To represent these results to a gene or complex level (in the form of a percentage), the number of changes per gene/complex were standardised based on gene size (i.e. total number of nonsynonymous changes/(gene size × total number of branches) × 100).

### Radical changes in physicochemical properties of amino acids

The impact of positive selection on physical and chemical properties of 31 amino acids was evaluated for each of the 13 PCGs using TreeSAAP v3.2 (Selection on Amino Acid Properties)^114^ by detecting observed changes inferred by the phylogenetic tree under neutral conditions. The impact of 20 physicochemical properties (excluding 11 amino acids with < 85% accuracy following McClellan and Ellison^115^) were evaluated. Under the assumption of neutrality, a significant positive z-score as calculated by TreeSAAP (z-score > 3.09, *p* < 0.001) for a physicochemical property is indicative of the prevalence of nonsynonymous substitutions, while a significant negative z-score (z-score < − 3.09, *p* < 0.001) indicates the prevalence of synonymous substitutions. A sliding window of 15 codons was utilized, based on a categorical scale of 1 (most conservative) to 8 (most radical). Only the most radical changes (category scores of 7 and 8, *p* ≤ 0.001) were considered, and for the physicochemical properties that were significant for both category 7 and 8, only the z-scores that were significant for category 8 were included.

## Supplementary Information

Supplementary Information 1.

Supplementary Information 2.

## Data Availability

The authors confirm that all relevant data used for this manuscript are available online from the GenBank NCBI online repository (https://www.ncbi.nlm.nih.gov/genbank/). All sequence accession numbers are list in the Supplementary Information of this manuscript (Table S1).
