# Supplementary material for: Mitogenome selection in the evolution of key ecological strategies in the ancient hexapod class Collembola
Source: Sci Rep. 2022 Aug 31;12:14810. doi: 10.1038/s41598-022-18407-1 (PMC9433435; doi:10.1038/s41598-022-18407-1)
Supplement: Supplementary file 2 — Supplementary Information 2. [file 41598_2022_18407_MOESM2_ESM.pdf]

Supplementary Figures and Tables for

Mitogenome selection in the evolution of key ecological strategies in the ancient hexapod class Collembola

Daniela M. Monsanto, Devon C. Main, Charlene Janion-Scheepers, Arsalan Emami-Khoyi, Louis Deharveng, Anne Bedos, Mikhail Potapov, Shilpa P. Parbhu, Johannes J. Le Roux, Peter R. Teske, Bettine Jansen van Vuuren*

*Corresponding author: Bettine Jansen van Vuuren, ORCID ID 0000-0002-5334-5358

Email: bettinevv@uj.ac.za

This PDF file includes:

Figures S1 to S4

Tables S1 to S4

Legend for Dataset S1

SI References

Other supplementary materials for this manuscript include the following:

Dataset S1

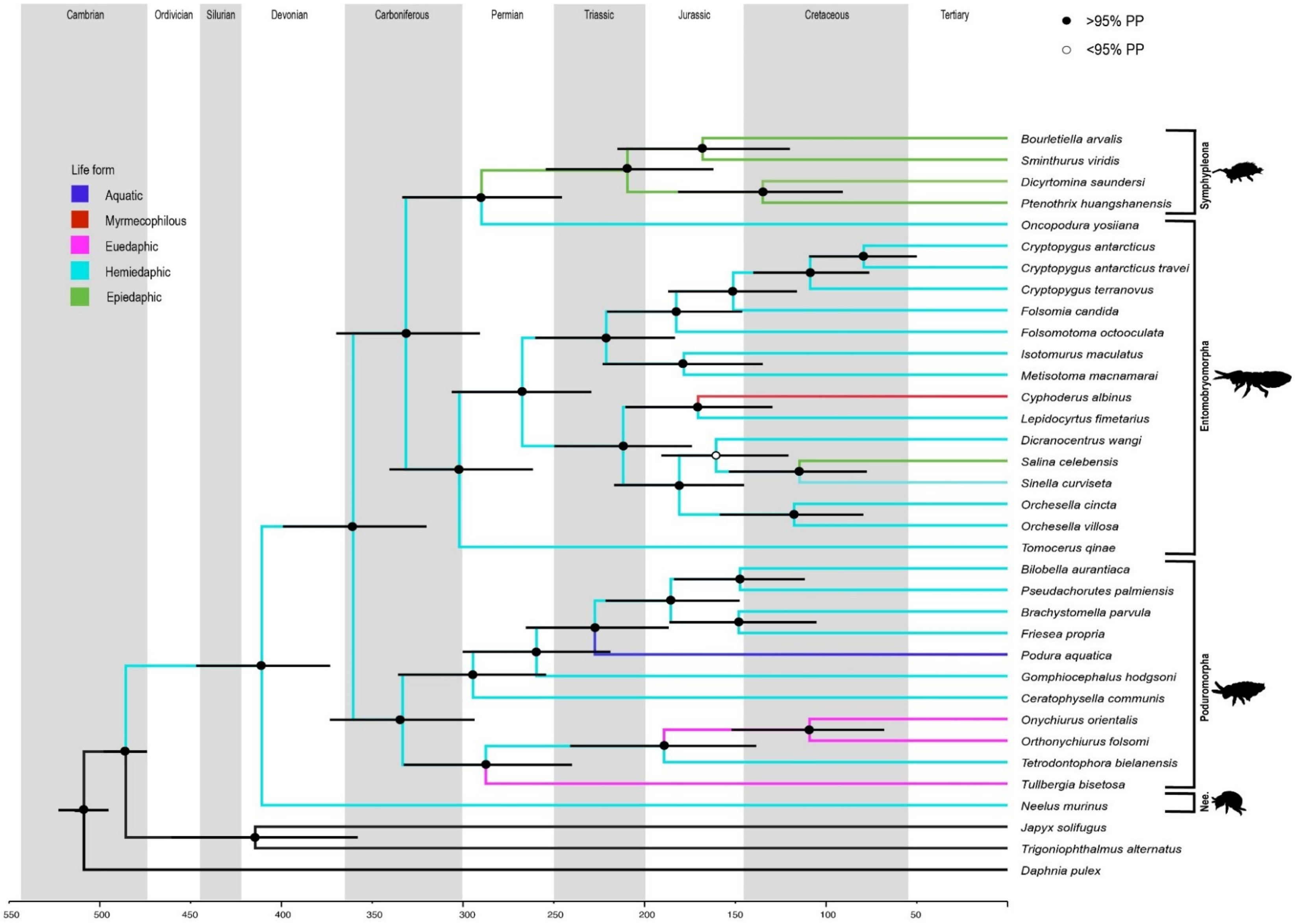
Cambrian
Ordivician
Silurian
Devonian
Carboniferous
Permian
Triassic
Jurassic
Cretaceous
Tertiary
>95% PP
<95% PP
Life form
Aquatic
Myrmecophilous
Euedaphic
Hemiedaphic
Epiedaphic
Bourletiella arvalis
Sminthurus viridis
Dicyrtomina saundersi
Ptenothrix huangshanensis
Oncopodura yosiiana
Cryptopygus antarcticus
Cryptopygus antarcticus travei
Cryptopygus terranovus
Folsomia candida
Folsomotoma octooculata
Isotomurus maculatus
Metisotoma macnamarai
Cyphoderus albinus
Lepidocyrtus fimetarius
Dicranocentrus wangi
Salina celebensis
Sinella curviseta
Orchesella cincta
Orchesella villosa
Tomocerus qinae
Bilobella aurantiaca
Pseudachorutes palmiensis
Brachystomella parvula
Friesea propria
Podura aquatica
Gomphiocephalus hodgsoni
Ceratophysella communis
Onychiurus orientalis
Orthonychiurus folsomi
Tetrodontophora bielanensis
Tullbergia bisetosa
Neelus murinus
Japyx solifugus
Trigoniophthalmus alternatus
Daphnia pulex
Symphypleona
Entomobryomorpha
Poduromorpha
Nee.
550
500
450
400
350
300
250
200
150
100
50

Figure S1. Dated Bayesian phylogeny based on the mitogenomes of 32 collembolan taxa from four orders (abbreviations Nee.: Neelipleona) and three outgroup taxa indicating the evolution of life forms for each species (blue – aquatic, red – myrmecophilous, pink – euedaphic, cyan – hemiedaphic, and green – epiedaphic). The blue bars indicate the 95% confidence interval for each divergence time estimate. Solid black node circles correspond to nodes with a Bayesian posterior probability of ≥ 0.95, while the open white circled corresponds to a posterior probability of < 0.95.

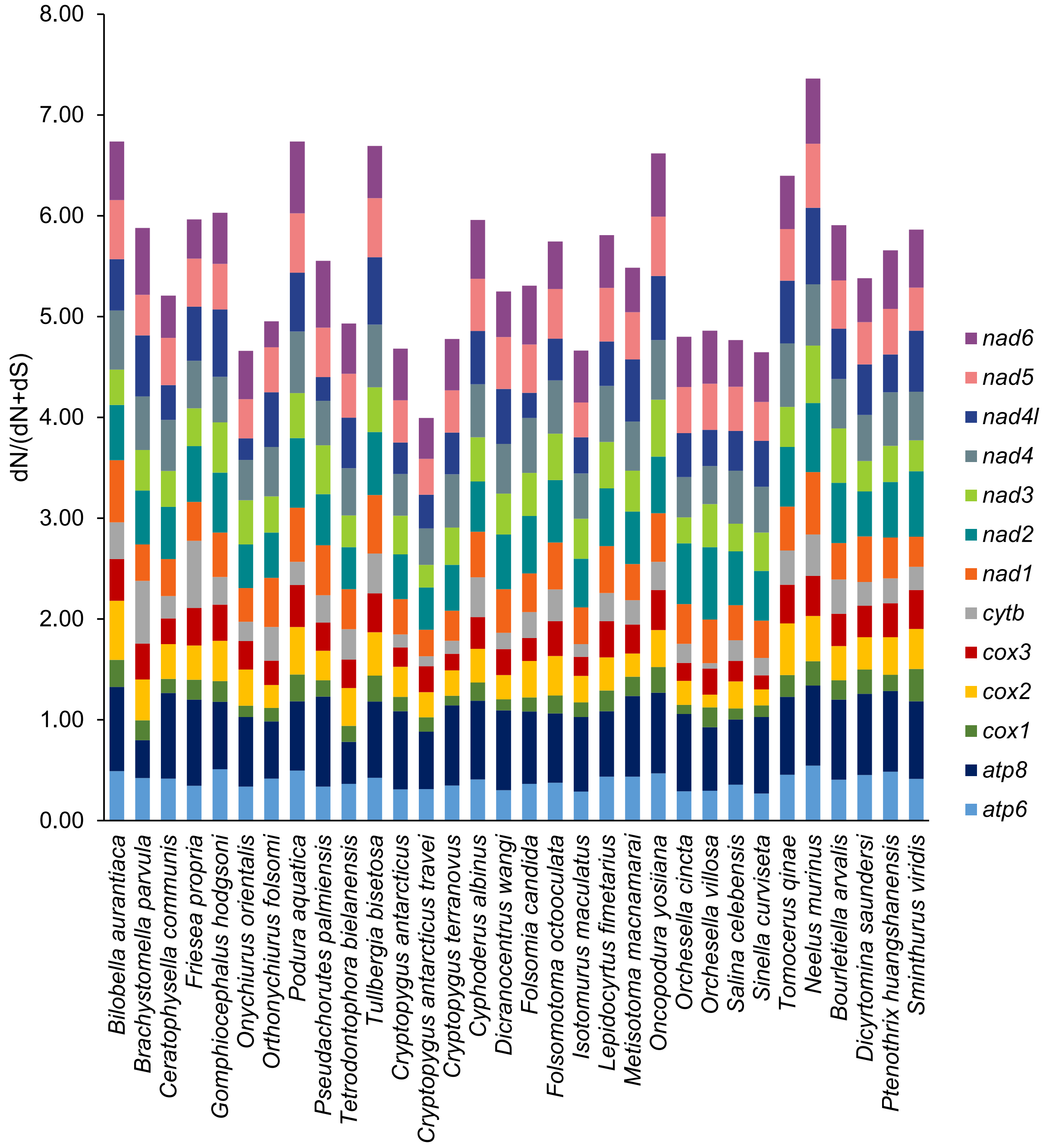

Figure S2. Stacked bar plots representing the number of nonsynonymous mutations as a proportion of the total mutations (i.e. dN/(dN+dS)) for each gene and taxon. Each gene and the corresponding proportion is represented by the size of each coloured stack, and the total size of the stacked column, corresponds to the sum of dN/(dN+dS) across the genes per taxon.

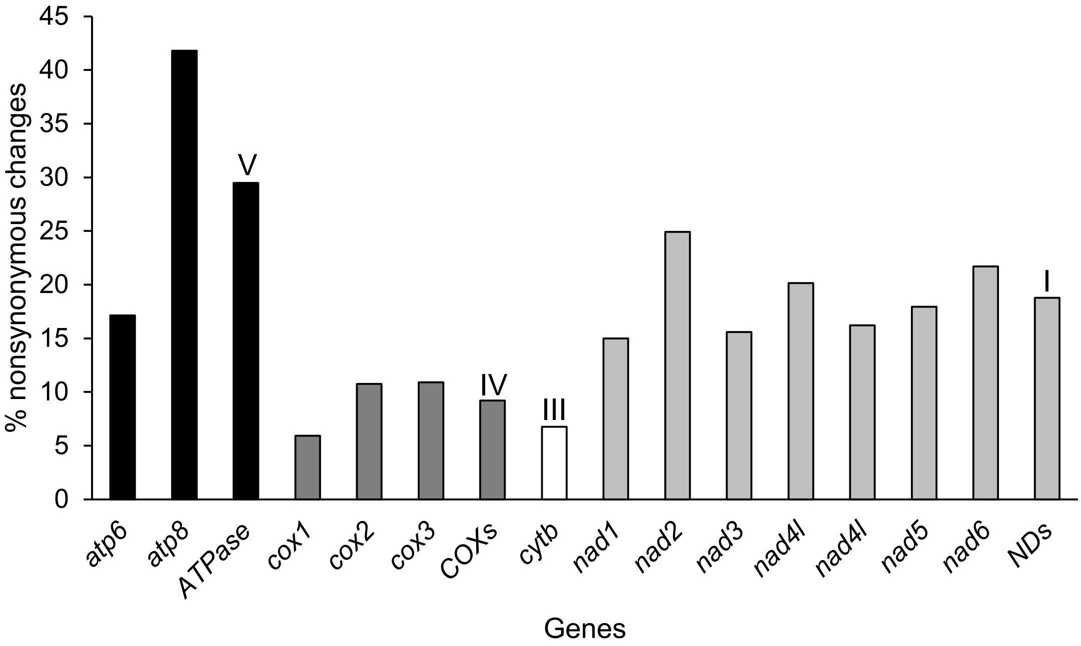

Figure S3. Bar chart showing the percentage of nonsynonymous mutations (standardised by gene size) that have occurred per gene. The letters above the columns refer to the mean of the nonsynonymous mutations for each of the four complexes.

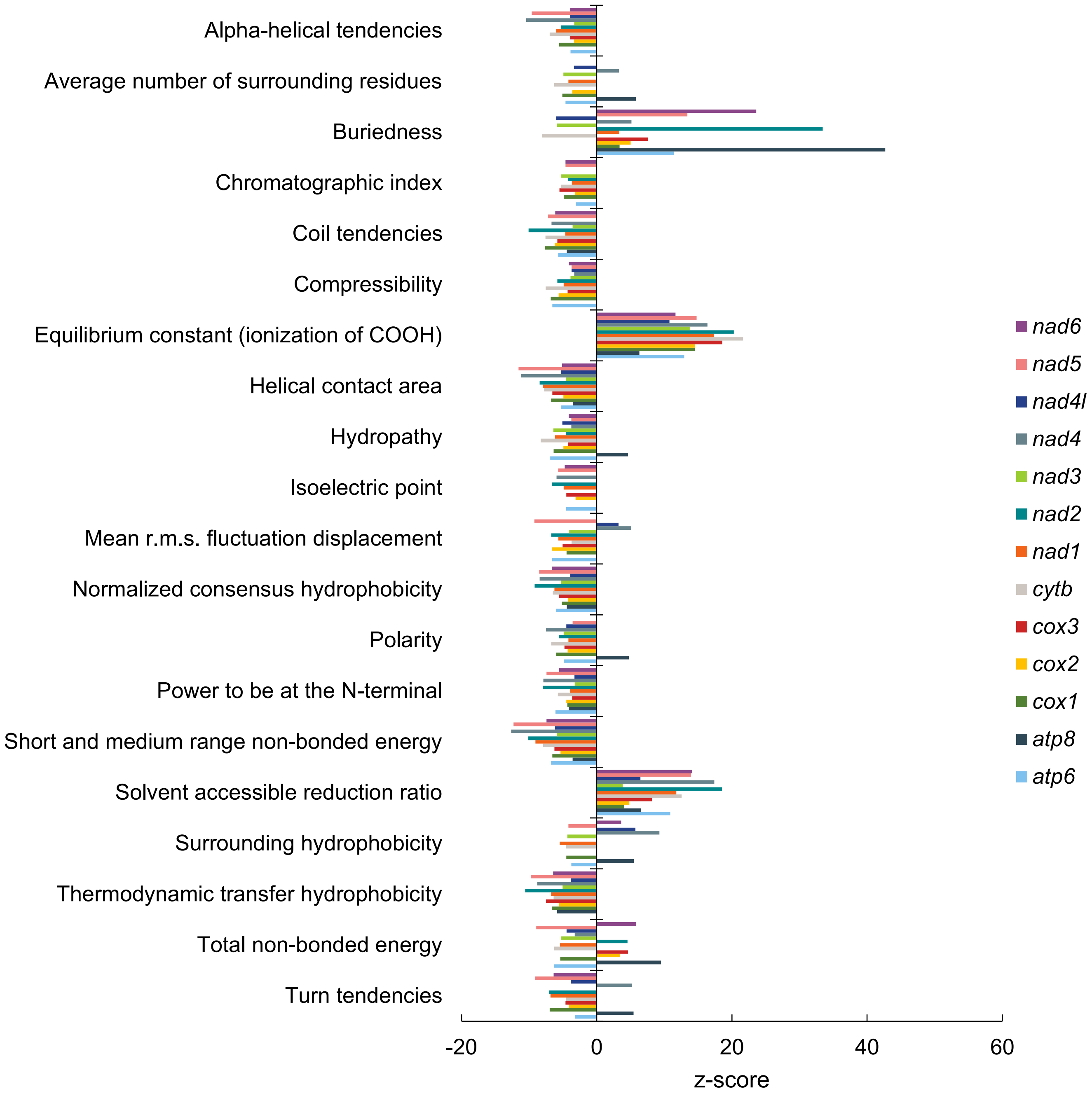

Figure S4. Significant positive and negative z-scores of the radical amino acid changes for 20 amino acid physicochemical properties as identified by the TreeSAAP analyses for each PCG (coloured bars). Positive z-scores represent positive/diversifying selection, while negative z-scores indicate negative/purifying selection.

Table S1. Details of mitogenomes of collembolan and outgroup taxa included in this study.

Accession number	Species	Authority	Order	Genome length (bp)
EU084034	*Bilobella aurantiaca*	(Caroli, 1912)	Poduromorpha	16,312
MN660050	*Brachystomella parvula*	(Schäffer, 1896)	Poduromorpha	15,002
NC_046523	*Ceratophysella communis*	(Folsom, 1898)	Poduromorpha	15,331
EU124719	*Friesea propria**	Carapelli et al., 2020	Poduromorpha	15,442
AY191995	*Gomphiocephalus hodgsoni*	Carpenter, 1908	Poduromorpha	15,075
NC_006074	*Onychiurus orientalis*	(Martynova, 1976)	Poduromorpha	12,984
MN661001	*Orthonychiurus folsomi*	(Schäffer, 1900)	Poduromorpha	15,283
NC_006075	*Podura aquatica*	Linnæus, 1758	Poduromorpha	13,809
MN660051	*Pseudachorutes palmiensis*	Börner, 1903	Poduromorpha	17,110
AF272824	*Tetrodontophora bielanensis*	(Waga, 1842)	Poduromorpha	15,455
MK520870	*Tullbergia bisetosa*	Börner, 1903	Poduromorpha	15,204
NC_010533	*Cryptopygus antarcticus*	Willem, 1901	Entomobryomorpha	15,297
MK433191	*Cryptopygus antarcticus travei*	Deharveng, 1981	Entomobryomorpha	15,743
NC_037610	*Cryptopygus terranovus*	(Wise, 1967)	Entomobryomorpha	15,352
NC_046888	*Cyphoderus albinus*	Nicolet, 1842	Entomobryomorpha	14,836
NC_046887	*Dicranocentrus wangi*	Ma & Chen, 2007	Entomobryomorpha	14,883
KU198392	*Folsomia candida*	Willem, 1902	Entomobryomorpha	15,147
KC862316	*Folsomotoma octooculata*	(Willem, 1901)	Entomobryomorpha	15,338
MK509021	*Isotomurus maculatus*	(Schäffer, 1896)	Entomobryomorpha	15,263
NC_047189	*Lepidocyrtus fimetarius*	Gisin, 1964	Entomobryomorpha	14,698
MN592792	*Metisotoma macnamarai*	(Folsom, 1918)	Entomobryomorpha	15,177
NC_046886	*Oncopodura yosiiana*	Szeptycki, 1977	Entomobryomorpha	14,808
NC_032283	*Orchesella cincta*	(Linnæus, 1758)	Entomobryomorpha	15,728
EU016195	*Orchesella villosa*	(von Linné, 1767)	Entomobryomorpha	14,924
NC_046522	*Salina celebensis*	(Schäffer, 1898)	Entomobryomorpha	14,788
NC_042755	*Sinella curviseta*	Brook 1882	Entomobryomorpha	14,840
MK423966	*Tomocerus qinae*	Yu, Yao & Hu, 2016	Entomobryomorpha	15,045
MH155200	*Neelus murinus*	Folsom, 1896	Neelipleona	13,992
NC_039558	*Bourletiella arvalis*	(Fitch, 1862)	Symphypleona	14,794
MG70139	*Dicyrtomina saundersi*	(Lubbock, 1862)	Symphypleona	15,045
MK423965	*Ptenothrix huangshanensis*	Chen & Christiansen, 1996	Symphypleona	15,152
EU016192	*Sminthurus viridis*	(Linnæus, 1758)	Symphypleona	14,817
NC_000844	*Daphnia pulex*	Leydig 1860	Diplostraca	15,333
NC_007214	*Japyx solifugus*	Haliday, 1864	Diplura	15,785
NC_010532	*Trigoniophthalmus alternatus*	(Silvestri 1904)	Archaeognatha	16,197

*Mitogenome sequence originally submitted as *Friesea grisea*, but was subsequently redescribed as *F. propria* [1].

Table S2. Maximum likelihood estimates of positive selection using the CodeML algorithm across the phylogeny for each gene for the site-based substitution models that had significant likelihood ratio test *p*-values (LRT *p*-value). Np: number of parameters, Ln L: log-likelihood values.

Gene	Model	np	Ln L	Model compared (null vs alt. models	LRT *p*-value	Positive selection sites
atp6	M7<br>M8	71<br>73	-15531.54<br>-15513.4	M7 vs M8	0.000000013	39 T 0.776, 41 I 0.698, 56 T 0.603<br>Not Allowed
atp8	M7<br>M8	71<br>73	-4573.41<br>-4563.13	M7 vs M8	0.000034	7 I 0.521<br>Not Allowed
	M8a<br>M8	72<br>73	-4553.72<br>-4563.13	M8a vs M8	0.000014	Not Allowed
cox1	M7<br>M8	71<br>73	-25376.1<br>-25355.2	M7 vs M8	0.000000001	Not Allowed
cox2	M7<br>M8	71<br>73	-12090.4<br>-12085.6	M7 vs M8	0.008	Not Allowed
cytb	M7<br>M8	71<br>73	15006.98<br>-14996.2	M7 vs M8	0.00002	Not Allowed
nad1	M7<br>M8	71<br>73	-17337.9<br>-17318.4	M7 vs M8	0.000000003	73 I 0.610, 138 I 0.944<br>Not Allowed
nad3	M8a<br>M8	72<br>73	-7268.23<br>-7277.19	M8a vs M8	0.000023	Not Allowed
nad4	M1a<br>M2a	71<br>73	-29584.3<br>-29581	M1a vs M2a	0.039	Not Allowed
nad4l	M7<br>M8	71<br>73	-5724.28<br>-5720.07	M7 vs M8	0.015	32 F 0.728<br>Not Allowed
nad6	M7<br>M8	71<br>73	-10506.9<br>-10495.4	M7 vs M8	0.000009965	77 T 0.572<br>Not Allowed
	M8a<br>M8	72<br>73	-10482.2<br>-10495.4	M8a vs M8	0.000000272	Not Allowed

Table S3. Number of nonsynonymous changes as a proportion of the total changes (i.e. dN/(dN+dS)) and ω = dN/dS for each terminal branch/taxon.

Terminal branch/taxon	Proportion (dN/(dN+dS))	ω = dN/dS
Bilobella aurantiaca	0.52	1.36
Brachystomella parvula	0.45	0.94
Ceratophysella communis	0.40	1.00
Friesea propria	0.46	1.21
Gomphiocephalus hodgsoni	0.46	0.99
Onychiurus orientalis	0.36	0.66
Orthonychiurus folsomi	0.38	0.69
Podura aquatica	0.52	1.27
Pseudachorutes palmiensis	0.43	1.32
Tetrodontophora bielanensis	0.38	0.65
Tullbergia bisetosa	0.51	1.25
Cryptopygus antarcticus	0.36	0.75
Cryptopygus antarcticus travei	0.31	0.49
Cryptopygus terranovus	0.37	0.81
Cyphoderus albinus	0.46	1.03
Dicranocentrus wangi	0.40	0.92
Folsomia candida	0.41	0.85
Folsomotoma octooculata	0.44	0.90
Isotomurus maculatus	0.36	0.72
Lepidocyrtus fimetarius	0.45	0.90
Metisotoma macnamarai	0.42	0.97
Oncopodura yosiiana	0.51	1.28
Orchesella cincta	0.37	0.80
Orchesella villosa	0.37	0.78
Salina celebensis	0.37	0.68
Sinella curviseta	0.36	0.74
Tomocerus qinae	0.49	1.16
Neelus murinus	0.57	1.61
Bourletiella arvalis	0.45	1.04
Dicyrtomina saundersi	0.41	0.92
Ptenothrix huangshanensis	0.44	1.00
Sminthurus viridis	0.45	1.03

Table S4. Number of nonsynonymous substitutions as a proportion of the total changes (i.e. dN/(dN+dS)) and ω = dN/dS for each gene.

Gene	Proportion (dN/(dN+dS))	ω = dN/dS
atp6	0.39	0.67
atp8	0.73	3.21
cox1	0.18	0.23
cox2	0.34	0.54
cox3	0.30	0.44
cytb	0.27	0.44
nad1	0.42	0.77
nad2	0.54	1.27
nad3	0.40	0.70
nad4	0.50	1.04
nad4l	0.48	1.06
nad5	0.47	0.93
nad6	0.52	1.16

Dataset S1 (separate file but with reference list included below). Details of the morphological and ecological characteristics of the collembolan taxa included in this study.

SI References

1. Carapelli, A. *et al.* Evidence for cryptic diversity in the 'Pan-Antarctic' springtail *Friesea antarctica* and the description of two new species. *Insects* **11**, 141 (2020).
2. Baird, H. P., Leihy, R. I., Scheepers, C. J. & Chown, S. L. The ecological biogeography of indigenous and introduced Antarctic springtails. *J. Biogeogr.* **46**, 1–15 (2019).
3. Bellinger, P. F., Christiansen, K. A. & Janssens, F. Checklist of the Collembola of the world. http://www.collembola.org (2021).
4. Caroli, E. Contribuzioni alla conosenza dei Collemboli italiani. I. La tribù degli Achorutini CB. (1906). *Achorutes aurantiacus* n. sp. *Arch. Zool. Ital.* **6**, 367–370 (1912).
5. Arbea, I. J. & Jordana, R. Familia Neanuridae. in *Fauna Iberica* (ed. Ramos, M. A.) 272–477 (Museo Nacional de Ciencias Naturales, CSIC, 1997).
6. Cassagnau, P. Les Collemboles Neanuridae des pays dinaro-balkaniques: leur intérêt phylogénétique et biogéographique. *Biol. Gall.* **8**, 185–203 (1979).
7. Porco, D. *et al.* Challenging species delimitation in Collembola: cryptic diversity among common springtails unveiled by DNA barcoding. *Invertebr. Syst.* **26**, 470–477 (2012).
8. Bendjaballah, M. *et al.* Annotated checklist of the springtails (Hexapoda: Collembola) of the Collo Massif, northeastern Algeria. *Zoosystema* **40**, 389–414 (2018).
9. Cassagnau, P. Le polymorphisme des chromosomes polytènes de *Bilobella aurantiaca* Caroli (Collemboles) et sa signification biogéographique et écologique. *Comptes rendus l'Académie des Sci.* **280**, 2777–2780 (1975).
10. Cassagnau, P. La variabilité des chromosomes polytènes chez *Bilobella aurantiaca*

Caroli (Collembola Neanuridae) et ses rapports avec la biogéographie et l'écologie de l'espèce. *Arch. Zool. expérimentale générale* **117**, 511–572 (1976).

11. Schäffer, C. *Die Collembola der umgebung von Hamburg und benachbarter gebiete. Jahrbuch der Hamburgischen Wissenschaftlichen Anstalten* (1896).

12. Fjellberg, A. *The Collembola of Fennoscandia and Denmark: Poduromorpha*. (Koninklijke Brill NV, 1998).

13. Weiner, W. M. & Najt, J. Species of *Brachystomella* (Collembola: Brachystomellidae) from the Neotropical region. *Eur. J. Entomol.* **98**, 387–413 (2001).

14. Babenko, A. B. *et al. Opredelitel' kollembol fauny SSSR [Identification Keys to collembolan genera of the USSR fauna] (in Russian)*. (1988).

15. Fjellberg, A. *The Collembola of Fennoscandia and Denmark, Part II: Entomobryomorpha and Symphypleona*. (Koninklijke Brill NV, 2007).

16. Gisin, H. Ökologie und lebensgemeinschaften der collembolen im schweizerischen exkursursiongebiet Basels. *Rev. Suisse Zool.* **50**, 131–224 (1943).

17. Folsom, J. W. Japanese Collembola Part I. *Bull. Essex Inst.* **29**, 51–57 (1898).

18. Greenslade, P., Ireson, J. & Skarżyński, D. Biology and key to the Australian species of *Hypogastrura* and *Ceratophysella* (Collembola: Hypogastruridae). *Austral Entomol.* **53**, 53–74 (2013).

19. Yosii, R. Studies on the Collembolan genus *Hypogastrura*. *Am. Midl. Nat.* **64**, 257–281 (1960).

20. Davidson, S. J. Mesofaunal responses to cattle dung with particular reference to Collembola. *Pedobiologia (Jena).* **19**, 402–407 (1979).

21. Wise, K. A. J. Collembola (springtails). *Antarct. Res. Ser.* **10**, 123–148 (1967).

22. Hayward, S. A. L., Worland, M. R., Convey, P. & Bale, J. S. Habitat moisture availability

and the local distribution of the Antarctic Collembola *Cryptopygus antarcticus* and *Friesea grisea*. *Soil Biol. Biochem.* **36**, 927–934 (2004).

23. Carpenter, G. H. Insecta Aptera. in *National Antarctic Expedition 1901–1904, Natural History* 1–5 (British Museum (Natural History), 1908).

24. Stevens, M. I. & Hogg, I. D. Long-term isolation and recent range expansion from glacial refugia revealed for the endemic springtail *Gomphiocephalus hodgsoni* from Victoria Land, Antarctica. *Mol. Ecol.* **12**, 2357–2369 (2003).

25. Meyer-Rochow, V. B., Reid, W. A. & Gal, J. An ultrastructural study of the eye of *Gomphiocephalus hodgsoni*, a collembolan from Antarctica. *Polar Biol.* **28**, 111–118 (2005).

26. Greenslade, P., Stevens, M. I., Torricelli, G. & D'Haese, C. An ancient Antarctic endemic genus restored: morphological and molecular support for *Gomphiocephalus hodgsoni* (Collembola: Hypogastruridae). *Syst. Entomol.* **36**, 223–240 (2011).

27. Martynova, E. F. Novye i maloizvestnye vidy fauny Sibiri [Species of the genus *Onychiurus* Gervais, 1841 (Collembola, Onychiuridae) of the North and North-East of Asia] (in Russian). *Novye maloizv Vidy Faun* **10**, 5–44 (1976).

28. Schäffer, C. Die arktischen und subarktischen Collembola. *Fauna Arctica* **1**, 237–258 (1900).

29. Arbea, J. I. & Kahrarian, M. New data on the Onychiurinae (Collembola: Poduromorpha) of Iran, with description of one new species. *Boletín la Soc. Entomológica Aragon.* **60**, 141–151 (2017).

30. Jordana, R., Arbea, J. I., Simon, C. & Lucianez, M. J. *Collembola Poduromorpha. Fauna Iberica* (Madrid: Museo Nacionalde Ciencias Naturales, 1997).

31. Weiner, W. M. Generic revision of Onychiurinae (Collembola: Onychiuridae) with a cladistic analysis. *Ann. la Société Entomol. Fr.* **32**, 163–200 (1996).

32. Arbea, J. I. & Jordana, R. Nota sobre la presencia masiva de *Onychiurus folsomi* Schaeffer (Collembola, Onychiuridae) en lechos de *Eisenia andrei* (Oligochaeta, Lumbricidae). *Bol. Sanid. Veg. Plagas* **14**, 535–540 (1988).

33. Greenslade, P. The potential of Collembola to act as indicators of landscape stress in Australia. *Aust. J. Exp. Agric.* **47**, 424–434 (2007).

34. Linnæus, C. *Systema Naturæ per Regna tria Naturæ, secundum Classes, Ordines, Genera, Species, cum Characteribus, Differentis, Synonymis, Locis., Tomus I, Editio Decima, Reformata, Holmiæ, (Laurentii Salvii)*. (1758).

35. Moen, P. & Ellis, W. N. Morphology and taxonomical position of *Podura aquatica* (Collembola). *Entomol. Gen.* **9**, 193–204 (1984).

36. Gisin, H. *Collembolen fauna Europas*. (Museum d'Histoire Naturelle, 1960).

37. Lubbock, J. Monograph of the Collembola and Thysanura. *Nature* **8**, 482 (1873).

38. Lubbock, J. Notes on the Thysanura - Part II. The Transactions of the Linnean Society of London, Vol. XXIII, Read June 19th. 589–601 (1862).

39. Hopkin, S. P. *Biology of the springtails (Insecta: Collembola)*. (Oxford University Press, 1997).

40. Pichard, S. The biology of *Podura aquatica* (Linné) Collembole. *Bull. Biol. Fr. Belg.* **107**, 292–299 (1973).

41. Sławska, M. & Sławski, M. Response of springtail communities (Hexapoda: Collembola) to attempts of clearcut silviculture improvement in Forest Promotional Complex 'Lasy Mazurskie' (in Polish). *Sylwan* **153**, 534–547 (2009).

42. Börner, C. Über neue altweltliche Collembolen, nebst Bemerkungen zur Systematik der Isotominen und Entomobryinen. in *Sitzungsberichte der Gesellschaft Naturforschender Freunde zu Berlin* 129–182 (1903).

43. Kaprus, I. & Weiner, W. M. The genus *Pseudachorutes* Tullberg, 1871 (Collembola, Neanuridae) in the Ukraine with descriptions of new species. *Zootaxa* **2166**, 1–23 (2009).

44. Arbea, J. I. & Jordana, R. The genus *Pseudachorutes* (Collembola, Neanuridae) from Navarra (Northern Iberian Peninsula), with description of a new species and a new subspecies. *Mitteilungen der schweizerischen Entomol. gesellschaft Bull. la société Entomol. suisse* **62**, 157–166 (1989).

45. Waga, M. Description d'un insecte aptère qui se trouve en quantité aux environs de Varsovie. *Ann. la société Entomol. Fr.* **11**, 264–272 (1842).

46. Krzysztofowicz, A. Postembryonic development of male reproductive system in *Tetrodontophora bielanensis* (Waga) (Collembola). *Acta Biol. Cracoviensia, Zool.* **10**, 289–299 (1967).

47. Kontschan, J., Muranyi, D. & Traser, G. Y. Data to the distribution of the *Tetrodontophora bielanensis* (Waga, 1842) (Collembola: Onychiuridae). *Ann. Hist. Musei Natl. Hyngarici* **95**, 107–111 (2003).

48. Dunger, W. Zur Kenntnis von *Tetrodontophora bielanensis* (Waga, 1842) (Collembola, Onychiuridae)., Abhandlungen und Berichte des Naturkundemuseums Görlitz, Band 37, Nr. 1. *1961* 79–99.

49. Chahartaghi, M., Maraun, M., Scheu, S. & Domes, K. Resource depletion and colonization: a comparison between parthenogenetic and sexual Collembola species. *Pedobiologia* **52**, 181–189 (2009).

50. Chernova, N. M., Potapov, M. B., Savenkova, Y. Y. & Bokova, A. I. Ecological significance of parthenogenesis in Collembola. *Entomol. Rev.* **90**, 23–38 (2010).

51. Janion-Scheepers, C. Collembola of South Africa. www.collembola.co.za (2021).

52. Greenslade, P. & Wise, K. A. Collembola of Macquarie Island. *Rec. Auckl. Inst. Museum*

23, 67–97 (1986).

53. Convey, P., Greenslade, P., Arnold, R. J. & Block, W. Collembola of sub-Antarctic South Georgia. *Polar Biol.* **22**, 1–6 (1999).

54. Willem, V. Les Collemboles recueillis par l'Expédition antarctique belge. Annales de la Société entomologique de Belgique, tome XLV, 3 X. 260–262 (1901).

55. Deharveng, L. Collemboles des Iles Subantarctiques de l'Ocean Indien. *Com. Natl. Fr. des Rech. Antarct.* **48**, 33–108 (1981).

56. Willem, V. Collemboles. Expédition antartique Belge, Zoologie. 1–19 (1902).

57. Nicolet, H. Recherches pour Servir á l'Histoire des Podurelles. *Nouv. Mem. la Soc. Helv. des Sci. Nat.* **6**, 1–88 (1842).

58. Soto-Adames, F. N. Four new species and new records of springtails (Hexapoda: Collembola) from the US Virgin Islands and Puerto Rico, with notes on the chaetotaxy of Metasinella and Seira. *Caribb. J. Sci.* **38**, 77–105 (2014).

59. Delamare Deboutteville, C. Collemboles de Madagascar (Première note). *Bull. la Société Entomol. Fr. Mars* 38–41 (1948).

60. Ma, Y. & Chen, J.-X. A new *Dicranocentrus* species (Collembola: Entomobryidae) from China with a key to all species in the genus from Asia. *Zootaxa* **1633**, 63–68 (2007).

61. Ratnasingham, S. & Hebert, P. D. N. BOLD: the barcode of life data system (http://www.barcodinglife. org). *Mol. Ecol. Notes* **7**, 355–364 (2007).

62. Cipola, N. G., Silva, D. D. & Bellini, B. C. Class Collembola. in *Thorp and Covich's Freshwater Invertebrates* (eds. Thorp, J. H. & Rogers, D. C.) 11–55 (Elsevier Inc., 2018).

63. Potapov, M. *Synopses on Palaearctic Collembola, Volume 3. Isotomidae., Abhandlungen und Berichte des Naturkundemuseums Görlitz, Band 73, Heft* 2. (2001).

64. Fanciulli, P. P., Leo, C., Convey, P., Frati, F. & Carapelli, A. Redescription and neotype designation of the Antarctic springtail *Folsomotoma octooculata* (Collembola: Isotomidae). *Zootaxa* **4392**, 392–400 (2018).

65. Carapelli, A., Frati, F., Fanciulli, P. P. & Dallai, R. Taxonomic revision of 14 south-western European species of *Isotomurus* (Collembola, Isotomidae), with description of four new species and the designation of the neotype for *I. palustris*. *Zool. Scr.* **30**, 115–143 (2001).

66. Gisin, H. Collemboles d'Europe. VI., Revue Suisse de Zoologie, Tome 71, no 20, Mai 1964. in 383–400 (1964).

67. Wang, F., Chen, J. & Christiansen, K. Taxonomy of the genus *Lepidocyrtus* s.l. (Collembola: Entomobryidae) in East and Southeast Asia and Malaysia, with description of a new species from the People's Republic of China. *Can. Entomol.* **135**, 823–837 (2003).

68. Folsom, J. W. A new *Isotoma* of the snow fauna. *Can. Entomol.* **50**, 291–292 (1918).

69. Potapov, M., Babenko, A., Fjellberg, A. & Schulz, H. Taxonomy of a predaceous springtail: the revision of the Palaearctic species of *Metisotoma* Maynard, 1951. *Zootaxa* **4399**, 69–86 (2018).

70. Szeptycki, A. North Korean Collembola. II. The genus *Oncopodura* Carl et Lebedinsky, 1905 (Oncopoduridae). *Acta Zool. Cracoviensia* **22**, 45–54 (1977).

71. Saitoh, S., Fujii, S. & Takeda, H. Effect of habitat structural complexity on collembolan communities. *Ecol. Res.* **29**, 81–90 (2014).

72. Stach, J. *The Apterygotan fauna of Poland in relation to the world-fauna of this group of insects. Tribe: Orchesellini. Kraków*. (1960).

73. Baquero, E., Ledesma, E., Gilgado, J. D., Ortuño, V. M. & Jordana, R. Distinctive Collembola communities in the Mesovoid Shallow Substratum: First data for the Sierra

de Guadarrama National Park (Central Spain) and a description of two new species of *Orchesella* (Entomobryidae). *PLoS One* **12**, 1–32 (2017).

74. Folsom, J. W., Tanquary, M. C., Ekblaw, W. E., Land, C. & Schiaffer, A. Collembola from the Crocker land expedition 1913-1917. *Bull. Am. Museum Nat. Hist.* **41**, 271–303 (1919).

75. von Linné, C. Systema Naturæ, Tom. I. Pars II., Editio Duodecima Reformata, Holmiæ, Impensis Direct. Laur. Salvii. 533–1327 (1767).

76. Schäffer, C. Die Collembola des Bismarck-Archipels nach der Ausbeute von Prof. Dr. F. Dahl., Archiv für Naturgeschichte, Vierundsechzigster Jahrgang, I. Band, 3. Heft. 393–425 (1898).

77. Oliveira, F. G., Cipola, N. & Almeida, E. Systematics and biogeography of *Salina* MacGillivray (Collembola: Entomobryoidea), with emphasis on the species groups in the New World. *Insect Syst. Evol.* **51**, 81–138 (2018).

78. Gruner, D. S., Taylor, A. D. & Forkner, R. E. The effects of foliar pubescence and nutrient enrichment on arthropod communities of *Metrosideros polymorpha* (Myrtaceae). *Ecol. Entomol.* **30**, 428–443 (2005).

79. Blackith, R. & Disney, R. Passive dispersal during moulting in tropical Collembola. *Malayan Nat. J.* **41**, 529–531 (1988).

80. Brook, G. Notes on some little-known Collembola, and on the British species of the genus *Tomocerus*. 19–26 (1882).

81. Waldorf, E. Variations in cleaning between the sexes of *Sinella coeca* (Collembola: Entomobryidae). *Psyche* **81**, 254–257 (1974).

82. Wei, Q. *et al.* First note on chromosomes and male meiosis division of Collembola in China. *Zool. Res.* **26**, 96–100 (2005).

83. Lee, B.-H. & Park, K.-H. Some Entomobryidae including six new species and one new record of cave form (Collembola) from Korea. *Korean J. Zool.* **27**, 177–188 (1984).

84. Bandow, C., Coors, A., Karau, N. & Rombke, J. Interactive effects of λ-cyhalothrin, soil moisture, and temperature on *Folsomia candida* and *Sinella curviseta* (Collembola). *Environ. Toxicol. Chem.* **33**, 654–661 (2014).

85. Yu, D., Yao, J. & Hu, F. Two new species of *Tomocerus ocreatus* complex (Collembola, Tomoceridae) from Nanjing, China. *Zootaxa* **1**, 125–134 (2016).

86. Folsom, J. W. *Neelus murinus*, representing a new thysanuran family. *Psyche* **7**, 391–392 (1896).

87. Bretfeld, G. *Synopses on Palaearctic Collembola, Vol. 2, Symphypleona. Staatliches Museum für Naturkunde, Görlitz.* (1999).

88. Marín, E. P. & Palacios-Vargas, J. G. Redescription of *Neelus fimbriatus* Bretfeld & Trinklein, 2000 (Collembola: Neelidae) from Colombia. *Zootaxa* **4527**, 414–424 (2018).

89. Schneider, C. Morphological review of the order Neelipleona (Collembola) through the redescription of the type species of *Acanthoneelidus*, *Neelides* and *Neelus*. *Zootaxa* **4308**, 1–94 (2017).

90. Kovac, L. & Papac, V. Revision of the genus *Neelus* Folsom, 1896 (Collembola, Neelida) with the description of two new troglobiotic species from Europe. *Zootaxa* **2663**, 36–52 (2010).

91. Fitch, M. D. Field flea, *Symnthurus arvalis*, new species. (Aptera. Poduridae.). in *Transactions of the New-York state agricultural society with an abstract of the proceedings of the county agricultural societies* 673–674 (1862).

92. Stach, J. *The Apterygotan fauna of Poland in relation to the world-fauna of this group of insects. Family: Sminthuridae*. Kraków. 1–287 (1956).

93. Dallai, R., Fanciulli, P. P., Carapelli, A. & Frati, F. Aberrant spermatogenesis and sex determination in Bourletiellidae (Hexapoda, Collembola), and their evolutionary significance. *Zoomorphology* **120**, 237–245 (2001).

94. Stach, J. *The Apterygotan fauna of Poland in relation to the world-fauna of this group of insects. Families: Neelidae and Dicyrtomidae*. Kraków. 1–113 (1957).

95. Heckman, C. W. Collembola. in *Encyclopedia of South American Aquatic Insects: Collembola* 1–408 (Kluwer Academic Publishers, 2001).

96. Liu, W. P. A., Janion, C. & Chown, S. L. Collembola diversity in the critically endangered Cape Flats Sand Fynbos and adjacent pine plantations. *Pedobiologia* **55**, 203–209 (2012).

97. Ardron, P. A. 'Aliens in inner space UK, OK!': the occurrence of diverse, established communities of exotic springtails (Collembola) in formal gardens and more widely in the British landscape. *Int. Urban Ecol. Rev.* **4**, 10–23 (2009).

98. Chen, J.-X. & Christiansen, K. A new species of *Ptenothrix* from China (Collembola: Dicyrtomidae). *Florida Entomol.* **79**, 586–591 (1996).

99. Lubbock, J. Notes on the Thysanura - Part III. The Transactions of the Linnean Society of London, Vol. XXIII, 295–306 (1868).

100. Ponge, J., Dubs, F., Gillet, S., Sousa, J. P. & Lavelle, P. Decreased biodiversity in soil springtail communities: the importance of dispersal and landuse history in heterogeneous landscapes. *Soil Biol. Biochem.* **38**, 1158–1161 (2006).
